# Comprehensive Characterization of Anti-HLA and Non-HLA Antibodies in Patients on Kidney Transplant Waiting List and Evaluation of Their Impact on Alloimmunization Risk and Dialysis Treatment

**DOI:** 10.3390/ijms252212103

**Published:** 2024-11-11

**Authors:** Aida Mujić Franić, Marko Lilić, Nataša Katalinić, Ljubica Glavaš-Obrovac

**Affiliations:** 1 Laboratory for Tissue Typing, Clinical Hospital Center Rijeka, 51000 Rijeka, Croatia; amujic3@gmail.com (A.M.F.); natasa.katalinic@medri.uniri.hr (N.K.); 2 School of Medicine, University of Mostar, 88000 Mostar, Bosnia and Herzegovina; 3 Faculty of Medicine, University of Rijeka, 51000 Rijeka, Croatia; 4 Department of Medicinal Chemistry, Biochemistry and Clinical Chemistry, Faculty of Medicine Osijek, Josip Juraj Strossmayer University of Osijek, 31000 Osijek, Croatia

**Keywords:** kidney transplantation, antibody, anti-HLA, non-HLA, alloimmunization, dialysis, transplant rejection, immunological risk

## Abstract

Alloimmunization remains a major obstacle to successful kidney transplantation, mainly due to the formation of anti-HLA antibodies. In recent years, non-HLA antibodies have emerged as additional immunologic factors that can potentially contribute to graft rejection. The aim of this study was to investigate the prevalence and specificity of both anti-HLA and non-HLA antibodies in patients with end-stage renal disease on a waiting list for kidney transplantation. Serum samples from 74 patients were analyzed using complement-dependent cytotoxicity and solid-phase assays. IgG anti-HLA antibodies were identified in 43.2% of participants, while IgG non-HLA antibodies were detected in 91.9%. The most frequent non-HLA antibodies included anti-ENO1 (28.4%), anti-FIBR1 (23.0%) and anti-PRKCZ (23.0%). A significant difference was found between the number of distinct IgG anti-HLA and IgG non-HLA antibody specificities. However, no significant correlation was found between the number of IgG non-HLA antibody specificities and previous alloimmunization events or dialysis treatments. These results suggest that non-HLA antibodies, although often overlooked, can sometimes play a critical role in transplant outcomes. Routine testing for non-HLA antibodies, in addition to mandatory anti-HLA antibody screening and identification, could improve immunologic risk assessment in transplant patients and post-transplant care.

## 1. Introduction

The immune system is a finely tuned network that adapts to various threats and maintains homeostasis in the body. Its main function is to distinguish between self and non-self by triggering immune responses against foreign molecules while maintaining tolerance to the body’s own antigens [1].

Of central importance for this function are the genes of the major histocompatibility complex (MHC), which is known in humans as the human leukocyte antigen (HLA). HLA molecules play a central role in transplantation medicine as well as in immune regulation, disease associations and various therapeutic applications such as reproductive immunology, transfusion medicine and forensic investigations [1]. The polymorphism of the HLA system, one of its main characteristics, with around 40,000 described alleles, ensures a diverse immune response but also poses a challenge in organ transplantation [2,3].

Organ transplantation is considered the optimal therapeutic approach for end-stage organ failure, including chronic kidney disease. Chronic kidney disease progresses to end-stage renal disease (ESRD) and requires renal function replacement therapy through dialysis or, preferably for a better patient quality of life, a kidney transplant [4]. Transplantation is considered the gold standard for treating end-stage organ failure. However, the immunological challenge posed by alloimmunization—the immune response to non-self HLA molecules—remains a significant obstacle to successful transplantation. It reduces the likelihood of finding a compatible donor and increases immunological risk, leading to poorer transplant outcomes [5,6,7,8,9,10]. Natural antibodies against A and B antigens of the ABO blood group system, that primarily are IgM, strongly activate complement and play a key role in the humoral immune response to transplants [11,12]. The incompatibility of the ABO blood group system and the HLA antigens represents a major immune risk in allogeneic transplantation. Alloimmunization usually arises from previous exposure to mismatched foreign HLA antigens through blood transfusions, pregnancy or previous transplants, which leads to the formation of anti-HLA antibodies [13,14]. These antibodies can mediate rejection by targeting mismatched HLA antigens on the graft cells, a process known as antibody-mediated rejection (ABMR) [14,15,16]. For decades, research has focused on understanding how mismatches of HLA antigens contribute to graft rejection and failure. To prevent acute or chronic graft rejection after transplantation, it is essential to minimize mismatches between the patient and the organ donor by regularly monitoring unacceptable antigen specificities in patients on a transplant waiting list. Traditionally, cytotoxicity-based assays have been used to assess the immunological risk in transplant recipients [15]. In clinical practice, this risk has been managed by regular monitoring for unacceptable antigen specificities in patients awaiting transplantation. This often involves determining the percentage of panel reactive antibodies (%PRA) directed against HLA antigens. A high %PRA poses a significant barrier to organ transplantation, and specific alloimmunization, determined by newer solid-phase assays, can render an organ completely unacceptable [17].

The clinical significance of HLA antibodies is well established in kidney transplantation. Antibodies against mismatched HLA class I and class II antigens, particularly donor-specific antibodies (DSAs), are strongly associated with acute and chronic rejection, delayed graft function (DGF) and graft loss [16]. Early studies showed that the presence of anti-HLA antibodies significantly increased the risk of graft failure, especially when DSAs were detected before transplantation [15,16]. In modern transplantation settings, the assessment of immune compatibility relies not only on conventional serological methods but more preferentially on advanced solid-phase assays that allow for the precise serum screening and identification of HLA-specific antibodies [15,18]. In addition, algorithms such as HLAMatchmaker and PIRCHE now help to minimize immune mismatch by predicting epitopic differences between donor and recipient HLA molecules [19,20]. Epitopes can be unique to a single HLA antigen or shared across several antigens. In the past, in serological testing, common epitopes were classified based on cross-reactive groups (CREGs) [21,22]. Technological advances have refined the definition of an epitope as a series of continuous short linear sequences, consisting of up to three polymorphic amino acid residues on the surface of the molecule, referred to as triplets. Subsequently, eplets—longer and often discontinuous sequences of polymorphic amino acids that are accessible to antibodies—were identified as key drivers in the induction of antibody responses. Notably, antigenicity (the ability to bind with an antibody) and immunogenicity (the ability to elicit an immune response) are crucial for the clinical relevance of these epitopes in transplantation [23,24]. These advances have led to a more detailed understanding of antibody–antigen interactions at the epitope level and have further improved the predictive power and accuracy of pre-transplant immunological evaluations [19,20].

While anti-HLA antibodies are known to be important contributors to graft rejection, there has been increasing evidence for almost two decades for the role of antibodies directed against antigens outside the HLA system (non-HLA) in the pathophysiology of transplantation outcomes [25,26]. Non-HLA antibodies have been implicated in various autoimmune diseases, and their presence in transplant recipients suggests that they contribute to graft rejection through different mechanisms than those mediated by anti-HLA antibody-mediated rejection [27,28]. These antibodies often target endothelial cells, receptors and structural proteins, including collagen and fibronectin, influencing both acute and chronic rejection [29,30]. Anti-endothelial cell antibodies (AECAs) and antibodies targeting the angiotensin II type 1 receptor (AT1R) have been associated with vascular inflammation and graft dysfunction, while anti-LG3 antibodies are associated with ischemia–reperfusion injury and delayed graft function [27,28,30,31]. The identification of specific non-HLA antibody targets, such as myosin, agrin and vimentin, has broadened the scope of transplant immunology beyond the traditional focus on HLA antigens [27]. For instance, antibodies against agrin, a structural protein in the glomerular basement membrane, are associated with chronic transplant glomerulopathy, while antibodies against type IV collagen and fibronectin are linked to transplant glomerulopathy and vascular rejection [27].

Non-HLA antibodies can be classified as alloantibodies, directed against polymorphic antigens that differ between recipient and transplant donor, or, more commonly, as autoantibodies that recognize self-antigens, which are usually cryptic [27]. These antibodies can be present before transplantation or may be generated de novo afterward [32]. Despite increasing evidence of their clinical relevance, non-HLA antibodies remain understudied in routine clinical practice. Although several commercial tests for the detection of non-HLA antibodies are available, routine screening is not yet standard practice in transplant medicine [28,31,33].

The exact mechanisms by which specific non-HLA antibodies function remain mostly unclear. In addition to complement activation, IgG antibodies can also mediate immune responses through opsonization and antigen neutralization, which contribute to an effective immune response [34]. Furthermore, IgG antibodies may exert immunomodulatory effects by either stimulating or suppressing inflammatory and immune processes. As studies continue to elucidate the mechanisms by which non-HLA antibodies contribute to graft rejection, it is becoming clear that these antibodies may act independently or synergistically with anti-HLA antibodies (DSA), enhancing immune responses against the graft and potentially leading to poorer outcomes [27,32,33,35,36,37].

The detection of non-HLA antibodies is becoming an important part of post-transplant monitoring as they can serve as biomarkers for the development of histological changes characteristic of graft dysfunction and possible rejection [32,37,38]. As research progresses, the role of non-HLA antibodies in predicting transplant outcomes is increasingly recognized, with potential implications for tailoring immunosuppressive therapies and improving graft survival. The emergence of commercial kits for non-HLA antibody testing in transplantation is encouraging from a translational standpoint. These kits can aid transplant programs in evaluating both non-HLA antibodies and HLA-DSA to better stratify immune risks post-transplantation [39,40].

The aim of this study was to investigate the specificity and characteristics of non-HLA antibodies and anti-HLA antibodies in patients with end-stage renal disease on a waiting list for kidney transplantation. We also wanted to determine the correlation between anti-HLA and non-HLA antibodies and to investigate the relationship between non-HLA antibodies and previous alloimmunization events and the type and duration of dialysis treatment.

## 2. Results

This study involved 74 participants on the waiting list for kidney transplantation at the Clinical Hospital Center (CHC) Rijeka in Rijeka, Croatia, including 50 men (67.6%) and 24 women (32.4%). The average age was 56.1 ± 12.8 years, with a median of 58 years. The youngest participant was 21 years old, and the oldest was 77 years old.

### 2.1. Presence of Anti-HLA Antibodies in the Study Ppulation

Using the CDC method, anti-HLA antibodies were detected in the sera of 21 participants (28.4%), as indicated by a %PRA greater than 3%. The remaining 53 participants (71.6%) were negative for anti-HLA antibodies by this method.

All sera were also tested for IgG anti-HLA antibodies using a solid-phase assay with microspheres. Out of the 74 sera tested, 39 (52.7%) were positive for IgG anti-HLA class I, class II or both, while 35 (47.3%) were negative.

A significant difference was observed between the frequencies of participants with anti-HLA antibodies detected by the CDC method and those detected by the solid-phase assay (*p* = 0.003), as determined by a two-sided *t*-test for two proportions.

### 2.2. Specificities of IgG Anti-HLA Antibodies in the Studied Population

The specificities of the IgG anti-HLA antibodies were determined for five clinically important HLA loci: HLA-A, -B, -C, -DRB1 and -DQB1, as well as the additional loci HLA-DRB3, -DRB4 and -DRB5. As shown in Table 1, Table 2, Table 3, Table 4 and Table 5, IgG anti-HLA antibodies were identified against almost all known HLA antigens, with the exception of HLA-B70, Cw11, Cw13 and DR53, against which no antibodies were detected.

The specificities of the IgG antibodies against HLA class I in the studied population are shown in Table 1, Table 2 and Table 3. The specificities of the IgG anti-HLA antibodies were determined in the sera of 32 participants (43.2%). No IgG anti-HLA antibodies were detected in the sera of the remaining 42 participants (56.8%). Anti-HLA class I antibodies were detected in the sera of 26 participants (35.1%) and anti-HLA class II antibodies in the sera of 21 participants (28.4%).

For certain antigenic specificities, results are given using broad antigens: HLA-B14 represents a broad antigen for B64 and B65; HLA-B15 for B62, B63, B71, B72, B75, B76 and B77; HLA-B40 for B60 and B61; HLA-Cw3 for Cw9 and Cw10. The frequencies of specific anti-HLA antibodies in the study population were compared with the frequencies of HLA alleles coding for the corresponding antigens in the Croatian population [38]. The study by Maskalan et al. [41] was conducted on a sample of 10,000 potential hematopoietic stem cell donors from the donor registry.

The specificities of the IgG anti-HLA class II antibodies are listed in Table 4 and Table 5. In particular, HLA-DR3 represents the broad antigen for DR17 and DR18. The study by Grubic et al. [42] was conducted on a sample of 210 unrelated individuals from Croatia.

### 2.3. Specificities of IgG Non-HLA Antibodies in the Studied Population

IgG non-HLA antibodies were detected in the sera of 68 participants (91.9%), while no antibody reactivity to any of the 60 non-HLA antigens in the panel was detected in the remaining 6 participants (8.1%).

A total of 605 IgG antibodies specific for non-HLA antigens were found in the studied population. The antibodies were formed against each of the 60 non-HLA antigens included in the panel. The non-HLA antigens present in the panel are listed in Supplementary Table S1. An IgG antibody to each non-HLA antigen in the panel was identified in the sera of at least 4 of the 74 participants. The highest frequency among the IgG non-HLA antibodies was observed for the anti-ENO1 antibody, which was detected in the sera of 21 participants (28.4%) and accounted for 3.5% of all detected IgG non-HLA antibodies in the present study. The absolute and relative frequencies of the 60 IgG non-HLA antibodies and their percentages of the total number of detected IgG non-HLA antibodies are shown in Table 6.

**Figure 1:**
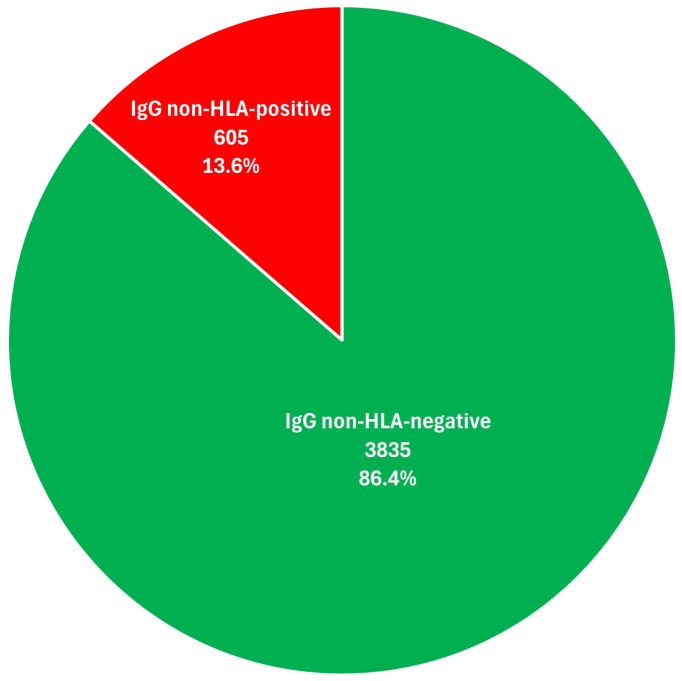
Distribution of reactivity of microsphere sets for the IgG non-HLA antibody determination in the studied population.

A total of 4440 data points were obtained for the entire study population by analyzing the reactivities of IgG non-HLA antibodies at the level of individual microsphere sets (74 participants × 60 non-HLA antigens in the LNHLA panel). The absolute and relative frequencies of reactivities for the microsphere sets in the study population are shown in Figure 1.

The distribution of the number of distinct IgG non-HLA antibody specificities per participant is shown in Table 7. In the studied population, the minimum number of different IgG non-HLA antibody specificities detected in a participant was 0, and the maximum was 59 (98.3% of the 60 antigens), with a mean of 8.2 and a median of 3. The majority of participants (43 of 74, 58.1%) tested positive for IgG antibodies against two to ten different non-HLA antigens.

In the sera of the largest number of participants, 39 (52.7%) IgG non-HLA antibodies were detected without the presence of IgG anti-HLA antibodies. Conversely, no IgG antibodies against either HLA or non-HLA antigens were detected in three participants (4.1%) (Table 8).

To assess the difference between the two groups of antibodies, a comparison was made between the number of specificities of each IgG anti-HLA and IgG non-HLA antibody (comparative analysis in Table 9, with descriptive statistics provided in Table 10). Using the Mann–Whitney U test, a significant difference (*p* < 0.001) was found between the IgG anti-HLA class I or II antibodies and IgG non-HLA antibodies.

### 2.4. Association of IgG Non-HLA Antibodies with Previous Alloimmunization Events

Most participants (55 of 74, 74.3%) had at least one history of alloimmunization. The transfusion of blood products accounted for the largest proportion with 49 participants (66.2%), including 35 men (47.3% of all participants) and 14 women (18.9% of all participants). An equal number of participants (18 out of 74, 24.3%) were exposed to alloimmunization due to pregnancy (corresponding to 75.0% of female participants) and a previous kidney transplantation. Among those awaiting a second kidney transplant, 15 were men (83.3% of this group) and 3 were women (16.7%). In addition, 14 participants (18.9% of the total; 58.3% of female participants) were both pregnant and received a transfusion of blood product.

Table 11 shows the distribution of participants according to their exposure to various alloimmunization events, including blood transfusion, pregnancy and prior kidney transplantation, and further stratifies them based on the presence or absence of IgG anti-HLA and IgG non-HLA antibodies. The comparison between participants with and without each antibody type (anti-HLA and non-HLA) shows significant differences for most alloimmunization events, except for anti-HLA antibodies in transfused patients, where no significant difference was found.

Based on the anamnestic data regarding transfusions of blood products, it was found that 49 participants (66.2%) had received a transfusion, while 25 participants (33.8%) had not. Comparing the mean values of the number of distinct non-HLA antibody specificities between these two groups revealed no significant difference (Table 12).

Of the 24 women in the study, 18 had been pregnant (75.0%), while 6 were not pregnant (25.0%). No significant difference was found in the mean values of the distinct specificities of the IgG non-HLA antibodies between the two groups (Table 13).

A point-biserial correlation coefficient was determined by statistical analysis of the number of IgG non-HLA antibody specificities with each of the alloimmunization events documented in the individual study participant’s medical history. No correlation was found between the number of IgG non-HLA antibody specificities and alloimmunization events in the studied population (Table 14).

### 2.5. Association of IgG Non-HLA Antibodies with the Method and Duration of Dialysis Treatment

In the studied population, 60 (81.1%) participants were undergoing dialysis treatment, including 40 men (66.7%) and 20 women (33.3%). Of these, 45 participants (75.0%) were treated with hemodialysis—32 (71.1%) men and 13 (28.9%) women—and 15 participants (25.0%) were treated with peritoneal dialysis—8 (53.3%) men and 7 (46.7%) women. Fourteen participants (18.9%) had not yet started dialysis treatment, including 10 men (71.4%) and 4 women (28.6%).

No significant difference in the number of distinct IgG non-HLA antibody specificities was found between the participants without dialysis treatment and those who underwent either type of dialysis treatment (Table 15).

The distribution of the duration of dialysis treatment at the time of serum sample collection is shown in Table 16. The largest group of participants on peritoneal dialysis were treated for 13 to 24 months (8.3%), with a median of 19 months, while the largest group of participants on hemodialysis were treated for 25 to 60 months (38.3%), with a median of 34 months. For all participants receiving dialysis treatment, the relative majority were treated for 25 to 60 months (38.3%), with a median of 30 months.

**Figure 2:**
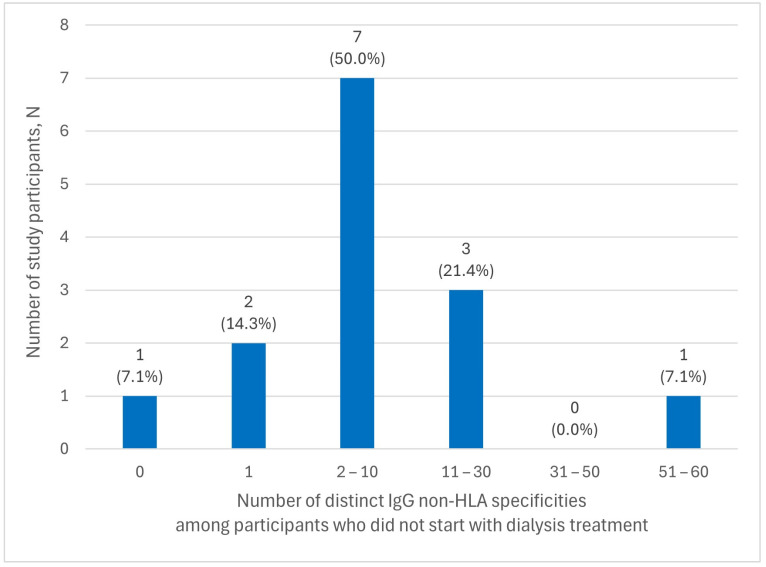
Distribution of the numbers of distinct IgG non-HLA antibody specificities among participants who did not start with dialysis treatment (N = 14).

Figure 2 shows the number of distinct IgG non-HLA antibody specificities in the sera of participants who had not started dialysis treatment at the time of serum sample collection. Most of these participants (7 of 14, 50.0%) had IgG antibodies against two to ten different non-HLA antigens.

Finally, Table 17 compares the number of distinct IgG non-HLA antibody specificities in participants who had started dialysis treatment (N = 60) versus those who had not (N = 14) at the time of serum sample collection (Figure 2). No significant difference was found between the two groups, regardless of the duration of treatment.

## 3. Discussion

Recent studies have reported graft rejection mediated by antibodies without detectable anti-HLA antibodies, suggesting that antibodies against molecules outside the HLA system (non-HLA) may play a significant role in graft survival outcomes [25,26,37,43]. Antibodies directed against mismatched HLA antigens can lead to shortened graft survival of the transplanted kidney, various forms of rejection, dysfunction and an increased incidence of side effects of immunosuppressive therapy. They arise as a result of patient exposure to foreign HLA antigens during prior transplants or other alloimmunization events, although they have also been detected in individuals without known alloimmunization [44,45]. Studies of various groups of patients on organ transplant waiting lists have shown that up to 60% of these patients have anti-HLA antibodies present [46,47]. This study included participants with an average age of 56.1 years suffering from ESRD. All participants were on the waiting list for a kidney transplant at CHC Rijeka in 2022.

### 3.1. Characterization of Anti-HLA Antibodies in the Studied Population

Serum testing using the CDC method detected anti-HLA antibodies in 28.4% of participants, indicating a potential immune risk. Solid-phase screening for anti-HLA antibodies revealed a significantly higher presence of IgG anti-HLA class I antibodies, anti-HLA class II antibodies or both in 52.7% of participants (*p* = 0.003). This difference indicates the higher sensitivity and specificity of solid-phase technology compared to the CDC method and is consistent with previous data [45].

In this study, the specificities of IgG anti-HLA antibodies were determined in the sera of 32 (43.2%) participants. The specificities of IgG anti-HLA class I antibodies were determined in 35.1% of participants, while IgG anti-HLA class II antibodies were found in 28.4% of participants. Only for the antigens HLA-B70, Cw11, Cw13 and DR53, no IgG anti-HLA antibody specificity was found. Several studies have previously investigated the frequency of HLA alleles and haplotypes in the Croatian population [41,42,45,48,49], with Tokić et al. providing an overview [50]. When comparing these studies with the frequencies of anti-HLA antibodies in the present population, anti-HLA-A2 was found to have the highest frequency (14.9%), while the allele group HLA-A*02 was the most frequent in Croatia (30.3%) [41], with a significant difference (*p* = 0.004). For HLA-B, anti-HLA-B15 antibodies had the highest frequency (67.6%), as HLA-B15 is a broad antigen that includes several antigens of the HLA-B locus (B62, B63, B71, B72, B75, B76 and B77). By grouping all antibodies against these antigens under the broad antigenic specificity HLA-B15, which is encoded by the alleles of the HLA-B*15 allele group, the frequency of anti-HLA-B15 antibodies increased, as some participants developed one or more specific antibodies against antigens within the B15 group. For the HLA-C locus, the common alleles encoding Cw4, Cw7 and Cw18 showed significantly lower antibody frequencies (*p* = 0.0226, *p* = 0.0002 and *p* ≤ 0.00001, respectively) [41]. For HLA-DR, anti-HLA-DR11 and anti-HLA-DR13 antibodies also showed lower frequencies, although the allele groups encoding these antigens are the two most common in the Croatian population [41]. A significant difference was observed between the frequency of alleles encoding HLA-DQ2 in the Croatian population (15.7%) and the anti-HLA-DQ2 antibody in the studied population (1.4%), *p* = 0.0011 [42]. In contrast, products from rare allele groups in the Croatian population, such as HLA-A*34, A*69, A*74 and A*80, elicited antibody responses more frequently (5.4% to 12.2%), probably due to greater immunogenicity, lower matching probability or both. The lower antibody frequencies against common HLA alleles could be explained by their prevalence in the population, which reduces the probability of HLA mismatch and consequently the development of alloantibodies after an alloimmunization event. Similar results regarding the relationship between HLA allele frequencies and specific anti-HLA antibodies have been described in previous studies [51,52].

Croatia has two EFI-accredited immunogenetics laboratories, at CHC Zagreb and CHC Rijeka, both of which are part of the Eurotransplant network. Each center has its own waiting list for kidney and other organ transplants. The distribution of HLA alleles and the formation of anti-HLA antibodies were examined in the patients on the waiting list at CHC Zagreb [53]. The highest frequencies of anti-HLA antibodies were against HLA-A24 (9.09%), A23 (7.93%), A25 (7.16%), B27 (4.71%), B44 (3.40%), Cw5 (12.24%), Cw2, Cw7 and Cw17 (9.18% each), DR1, DR4, DR9 and DR15 (8.55% each), DQ9 (16.31%) and DQ8 (15.80%) [53]. The results for anti-HLA-A were similar in the present study, with anti-HLA-A24, A25 and A23 (together with A32 and A69) following anti-HLA-A2 in frequency. For anti-HLA-B, however, there was no agreement between the two studies. While anti-HLA-Cw5 was the most frequent anti-HLA-C antibody in Zagreb, it had a low frequency (1.4%) in the present study. A high frequency of anti-HLA-DR15 and DR1 was found in both studies, although DR4 and DR9 were less common in the studied population.

### 3.2. Specificities of Non-HLA IgG Antibodies in the Studied Population

The most substantial aspect of this study was to determine the presence and specificity of IgG antibodies directed against non-HLA antigens in patients on the waiting list for a kidney transplant. Although allogeneic graft rejection in ABMR is usually caused by HLA-DSA, pathologic changes also occur in patients in whom circulating HLA-DSA are not present, suggesting that antigens outside the HLA system may contribute to graft rejection [18,27,32,37,54,55]. Although non-HLA antibodies have been studied extensively recently, the mechanisms of their generation and function in graft rejection are not yet fully understood [56,57,58]. Several recent studies have emphasized the clinical importance of non-HLA antibodies produced against modified alloantigens, which can trigger the alloimmune responses and the subsequent rejection of kidney transplants [59,60,61]. A synergistic effect between HLA-DSA and non-HLA antibodies leading to shortened graft survival has also been suggested [32,33,35,36,37,62,63].

The detection of 605 distinct IgG non-HLA antibodies in the entire study population emphasizes the extensive immune response to non-HLA antigens in the study population, with 13.6% of the total microsphere sets showing reactivity. Each non-HLA specificity was identified in at least four participants, demonstrating that non-HLA antibodies are not uncommon in ESRD patients. This study revealed a high prevalence of IgG non-HLA antibodies in the sera of patients on the kidney transplant waiting list, and 91.9% of participants showed reactivity to some of the 60 non-HLA antigens in the test panel. These results are consistent with the study by Shin et al., which demonstrated similar frequencies of non-HLA antibodies in patients with ESRD awaiting kidney transplantation—of the 32 non-HLA antigens from the panel studied, 0.4% to 97.1% of antibodies to these antigens were present in a single patient [64]. In another study by Burek Kamenaric et al., non-HLA antibodies were found in 93.9% of 33 highly sensitized patients (%PRA > 95%) on the kidney transplant waiting list in Zagreb, Croatia [65].

Among the non-HLA antibodies detected, anti-ENO1 was the most common with 28.4% of participants (corresponding to 3.5% of all antibodies detected). The high frequency of anti-ENO1 suggests that anti-ENO1 may play a role in non-HLA-mediated immune responses in these patients. Other common antibodies were anti-FIBR1 and anti-PRKCZ, which were each detected in 23.0% of participants. The detection of these antibodies in a significant proportion of the studied population raises concerns about their possible role in transplant outcomes, particularly their involvement in graft rejection. In the study by Burek Kamenaric et al., anti-ENO1 was also the most common non-HLA antibody (25.6%), followed by anti-STAT6 (16.3%) and a group of other antibodies, including GSTT1, IFNG, PLA2R1 and PRKCZ, each found in 13.9% of participants [65]. In the present study, all of these antibodies had frequencies greater than 10%. ENO1, a glycolytic enzyme associated with inflammatory responses and autoimmunity, may contribute to the immune system dysregulation observed in ESRD patients [66,67]. FIBR1, which is involved in blood clotting and tissue repair [68], and PRKCZ, a protein kinase associated with immune signaling, may enhance inflammatory and immune processes [69,70]. Anti-STAT6 antibodies have also been associated with ABMR in the absence of HLA-DSA [71].

The presence of non-HLA antibodies targeting structural proteins such as collagen and fibronectin, as well as endothelial markers such as ICAM1 and ROR1, suggests the impact on their interaction with the graft vasculature and other tissues that may contribute to dysfunction. Their involvement in endothelial injury and immune activity, particularly in the context of ischemia–reperfusion injury, should be further investigated as these processes are closely associated with graft outcomes in transplantation [27,28,29,30].

All 60 non-HLA antigens tested in this study were targeted by antibodies, confirming the broad spectrum of non-HLA immunization in ESRD patients. The fact that each antigen showed reactivity in at least four participants suggests a broad immune response by non-HLA antigens, possibly related to the chronic inflammation typical of ESRD [72].

Most participants (58.1%) had between two and ten distinct non-HLA antibody specificities, illustrating the complexity of their immune responses. This result mirrors the findings of Shin et al. in which most participants also had two to ten non-HLA specificities [64]. However, in the study by Burek Kamenaric et al., 69.7% of participants had one to five different non-HLA antibodies, while the remainder had six to 22 specificities [65]. Multiple antibody specificities could increase the immunogenic potential of total IgG non-HLA antibodies to trigger post-transplant alloimmune responses, possibly contributing to graft rejection. However, the exact clinical significance of multiple non-HLA antibody specificities remains to be fully elucidated. Adjusting immunosuppressive therapy based on specific non-HLA antibodies requires detailed clinical studies focusing on each antibody specificity. While this is an important area of investigation, it was beyond the scope of our current study, which aimed to assess the specificity and frequency of non-HLA antibodies in our patient population. Our findings offer a basis for future studies to explore the clinical implications of non-HLA antibodies on immunosuppressive therapy and transplant outcomes.

Interestingly, 8.1% of the participants in the present study showed no reactivity to non-HLA antigens. These individuals may have a particular immunologic profile or genetic predisposition that limits the production of non-HLA antibodies, which could reduce their risk of ABMR. Their absence of non-HLA antibodies could also be due to a more tolerant immune system or limited exposure to cryptic antigens that normally emerge during inflammatory or alloimmunization processes [27].

The lack of significant correlations between the number of non-HLA antibody specificities and traditional alloimmunization events—such as transfusion, pregnancy or prior transplantation—raises important questions about the factors that drive the development of non-HLA antibodies. This finding suggests that the formation of non-HLA antibodies follows different pathways than anti-HLA antibodies, possibly influenced by other factors such as autoimmune processes, chronic inflammation or even genetic factors that are not yet fully understood for each non-HLA antigen.

The three participants (labeled A, B and C here), two women (A, C) and one man (B), with the highest number of IgG non-HLA antibody specificities (51 to 60), were of particular interest for the comprehensive analysis. All three had no anti-HLA antibodies and had not previously undergone kidney transplantation. Of the 60 non-HLA antigens in the tested panel, participant A’s serum showed reactivity for 57 specificities (except for collagen I, collagen II and PRKCZ), participant B’s serum for 59 specificities (except for collagen I) and participant C’s serum for 58 specificities (except for collagen I and collagen V). Interestingly, collagen I was negative in all three patients with the highest number of antibody specificities. Participant A had no documented alloimmunization event, participant B had received a blood transfusion, and participant C had a history of pregnancy and transfusion. Participant A had been on peritoneal dialysis for 12 months, and participant C had been on hemodialysis for 34 months, while participant B was not receiving dialysis.

### 3.3. Correlation Between IgG Anti-HLA and IgG Non-HLA Antibodies

Due to the exceptional diversity and distribution of anti-HLA antibody specificities-and the correspondingly small number of participants with a certain specificity, it was not possible to draw relevant conclusions about possible correlations between the individual IgG anti-HLA and IgG non-HLA antibody specificities.

In the studied population, 3 out of 74 participants (4.1%) developed neither anti-HLA nor non-HLA antibodies. One of these participants had no documented alloimmunization events, while the other two had documented alloimmunization events (blood transfusion in both and an additional pregnancy in one). It is possible that these participants are unable to elicit an antibody-mediated immune response (non-responders). An equal number of participants, 3 of 74 (4.1%), produced only IgG anti-HLA without IgG non-HLA antibodies. In this group, one participant had three documented alloimmunization events (blood transfusion, pregnancy and previous transplantation), another had two events (blood transfusion and previous transplantation) and the last had one event (blood transfusion).

Of particular note were two male participants without documented alloimmunization events who had both anti-HLA and non-HLA antibodies. Previous studies have reported the presence of anti-HLA antibodies in men who had neither received blood transfusions nor undergone transplantation [73,74,75]. These natural anti-HLA antibodies are most likely formed due to cross-reactivity of HLA antigen epitopes with environmental antigens, such as those from food or microorganisms [76,77]. In the serum of one participant, a weak but definite positive specificity for HLA-A3 was detected. This antigen belongs to the CREG 1C, which includes HLA-A1, A3, A11, A23, A24, A29, A30, A31, A36 and A80 [78]. Interestingly, the study by Morales-Buenrostro et al. in a Mexican population identified HLA specificities from this CREG as the most common in men without a history of alloimmunization events [74]. In the serum of this participant, 14 different IgG non-HLA antibody specificities were identified. In the serum of the second participant, anti-HLA-DR52 and anti-STAT6 antibodies were observed.

Among the 68 out of the 74 participants (91.9%) who developed IgG non-HLA antibodies, 29 (39.2%) also developed IgG anti-HLA antibodies, while 39 (52.7%) had no IgG anti-HLA antibodies. This finding may have clinical implications that need to be determined in future studies for each non-HLA antigen. As previously discussed, the synergistic effect of anti-HLA and non-HLA antibodies has been observed to increase the risk of graft rejection [62,63]. It is, therefore, important to take this into account during post-transplant care.

According to a study by Senev et al. [37], patients who have only non-HLA antibodies (and no anti-HLA antibodies) in their serum still have some risk of graft rejection after transplantation. These patients require increased vigilance in monitoring both before and after transplantation to allow for possible adjustment of immunosuppressive therapy. The results of the present study are consistent with the research of Zhang et al. [35], who concluded that antibodies directed against antigens outside the HLA system are part of the general immune response in transplant rejection. These antibodies are often detected in patients with suspected ABMR even without HLA-DSA. The authors argue for the need to determine non-HLA antibodies in patients at high risk of rejection. The inclusion of testing for non-HLA antibodies in immunologic risk assessment could also have an impact on treatment protocols for graft rejection [65].

To quantify the degree of alloimmunization in individual participants who developed an immune response (responders), the number of specificities of IgG anti-HLA and IgG non-HLA antibodies was analyzed. The Mann–Whitney U test was used to compare the median numbers of specificities of each antibody group. A significant difference was found between the number of IgG non-HLA antibodies and IgG class I and class II anti-HLA antibodies.

### 3.4. Association of Anti-HLA and Non-HLA Antibodies with Previous Alloimmunization Events

Based on the medical history data, transfusions of blood products were identified as the cause of alloimmunization in 66.2% of the participants, most of whom were male. Pregnancy was considered the cause of alloimmunization in 75.0% of female participants, while the combination of transfusion and pregnancy occurred in 54.2% of female participants.

A significant difference was found between participants with and without anti-HLA antibodies, regardless of previous alloimmunization events, with the exception of participants exposed to blood transfusions. Two individuals (10.5%) who developed anti-HLA antibodies without having been exposed to alloimmunization have been reported previously. These results suggest that pregnancy, either alone or in combination with blood transfusion, and previous transplantation are key factors in stimulating anti-HLA antibody responses in the studied population. Similarly, a significant difference was observed between participants with and without non-HLA antibodies, independent of alloimmunization events. A limitation of the analysis was the small group of only six IgG non-HLA antibody-negative participants. Of note, even among participants who were not exposed to alloimmunization events, 94.7% (18/19) developed IgG non-HLA antibodies. This finding may be partially explained by the autoimmune nature of many non-HLA antibodies [79,80]. Therefore, the presence of non-HLA autoantibodies or undocumented alloimmunization events could explain this observation.

In the study population, there was no significant difference in the number of IgG non-HLA antibody specificities between participants who had received blood transfusions and those who had not (*p* = 0.683). There was also no significant difference when comparing participants who were pregnant with those who were not (*p* = 0.083). However, due to the small number of participants (N = 24), especially those who were not pregnant (N = 6), it may have been restricted to detect a significant difference between these groups. For the same reason, it is possible that the participants who were not pregnant had a higher mean number of non-HLA antibody specificities (19.71) than the participants who were pregnant (7.89).

Statistical analysis revealed no significant correlation between the number of non-HLA antibody specificities and any alloimmunization event (pregnancy, transfusion of blood products or transplantation). The correlation coefficients showed that there was no correlation in the study population. However, we cannot state with certainty that there is no correlation in the wider population, as the lack of statistical significance leaves room for the possibility that a correlation could exist but remained undetected in the present study. It is possible that, in this case, significance was not achieved in the studied population due to the small number of participants.

Regarding the type of dialysis treatment, no significant difference was found in the number of distinct IgG non-HLA antibody specificities between participants with and without dialysis treatment, regardless of whether it was peritoneal dialysis or hemodialysis. Similarly, no significant difference was found when comparing participants on peritoneal dialysis with those on hemodialysis, nor when comparing all three groups together. The observed lack of correlation for all monitored alloimmunization events suggests that the development of non-HLA antibodies may be influenced by individual immune response parameters and genomic characteristics and not solely by exposure to alloimmunization events or the type of dialysis treatment.

One of the parameters monitored was the duration of dialysis treatment prior to serum sampling. Participants on hemodialysis were mostly treated between 25 and 60 months (23/45), with a median of 34 months, while participants on peritoneal dialysis were mostly treated between 13 and 24 months (5/15), with a median of 19 months.

Regardless of the duration or type of dialysis, the most common range for the number of different IgG non-HLA antibody specificities was between two and ten. In both the overall dialysis group and the hemodialysis group, an increase in the number of participants with no or one IgG non-HLA antibody specificity to those with two to ten specificities was observed regardless of the duration of treatment. In these groups, a sharp decrease in the number of participants with 11 or more specificities was observed, also independent of treatment duration. This trend is consistent with the findings of Shin et al. [64]. A possible explanation for this is that it is less likely to find individuals with more than 10 different HLA specificities and that this pattern persists regardless of the duration of dialysis treatment. In addition, the likelihood of a patient remaining on dialysis decreases over time, as some will eventually undergo transplantation. Therefore, it is not surprising that the total number of patients with a long duration of treatment and more than 10 non-HLA specificities is very low.

In contrast, no consistent pattern was observed in patients receiving peritoneal dialysis. Instead, there were alternating increases and decreases in the number of participants over different treatment durations, while the number of non-HLA specificities increased. This irregularity is likely due to the small sample size, as the maximum number of participants in the peritoneal dialysis group was only three (two to ten non-HLA specificities and a treatment duration of 13 to 24 months).

## 4. Materials and Methods

### 4.1. Participants

This study was conducted with serum samples from patients who were on the kidney transplant waiting list at the CHC Rijeka in Rijeka, Croatia, in 2022. In that year, a total of 81 patients were registered on the kidney transplant waiting list, and 74 adult patients who met the inclusion criteria were enrolled in the study. Serum samples collected before the expected kidney transplantation were tested at least twice a year for the presence and specificity of anti-HLA antibodies, using both CDC and solid phase assays. At CHC Rijeka, this procedure is routinely performed every three months for patients on the waiting list. For the current study, serum samples from 2022 and available data on previous alloimmunizations documented in each participant’s medical history were used. For non-alloimmunized participants, the last serum sample that met the inclusion criteria was selected.

All participants’ serum samples were tested for the presence of anti-HLA and non-HLA antibodies. The tests were performed at the Laboratory for Tissue Typing, Clinical Institute for Transfusion Medicine, at CHC Rijeka, according to Eurotransplant guidelines, as Croatia is a member of Eurotransplant.

Inclusion criteria: Participants were included if they met the following criteria:

- Age > 18 years.
- Serum samples had been tested at least twice per year using the CDC method and solid-phase assay at CHC Rijeka.
- Available data on prior alloimmunization events.

Exclusion criteria: Patients (N = 7) were excluded if they

- Were tested fewer than twice per year, or only partially tested (e.g., not using all methods).
- Were under the age of 18.
- Lacked relevant patient history data.
- Had been partially tested at another center.
- Had died during 2022.

No patient was excluded from this study based on their previous medical history.

As this was anonymous retrospective research, participant consent was not required. The study was approved by the Ethics Committee of CHC Rijeka (Approval No.: 2170-29-02/1 22 2, 19 August 2022). Data were collected in accordance with fundamental ethical and bioethical principles, including personal integrity (autonomy), justice, beneficence and non-maleficence, as well as the Nuremberg Code and the latest revision of the Declaration of Helsinki. Confidentiality of the data obtained during the research was ensured.

### 4.2. Testing Methods

#### 4.2.1. Complement-Dependent Lymphocytotoxicity

Patient sera were tested for the presence of anti-HLA antibodies by the complement-dependent lymphocytotoxicity (CDC) assay, with a panel of lymphocytes carrying known HLA antigens from 50 different donors. The panel was designed to reflect the HLA antigen composition and frequency in the Croatian population. Results were read and recorded as the percentage of panel-reactive antibodies (%PRA), indicating the degree of alloimmunization. A serum sample with a CDC test result of %PRA > 3% was considered positive.

#### 4.2.2. Solid-Phase Assays

All participating histocompatibility and immunogenetics laboratories of Eurotransplant member states are required to conduct anti-HLA antibody testing using sera pretreated with ethylenediaminetetraacetic acid (EDTA). For this purpose, 38 µL of serum was combined with 2 µL of 6% EDTA solution after centrifugation.

All solid-phase assays used in this study were analyzed using a Luminex 200 instrument (Luminex/DiaSorin, Saluggia, Italy) and evaluated with MATCHIT! Antibody software, version 1.5 (Werfen/Immucor GTI Diagnostics, Waukesha, WI, USA).

##### Serum Testing for IgG Anti-HLA Antibodies by Solid-Phase Assays

The solid-phase assays were used to test for anti-HLA antibodies at two levels.

The first level involved screening for the presence or absence of IgG anti-HLA antibodies, using the Lifecodes Lifescreen Deluxe kit (Werfen/Immucor GTI Diagnostics) according to the manufacturer’s instructions. The results were expressed qualitatively as positive or negative for anti-HLA class I, class II or both.

For samples that tested positive in the screening assay, a second level of testing was performed to identify, i.e., determine the specificity of anti-HLA class I and II antibodies using the Lifecodes Single Antigen (LSA) assays for HLA class I, class II or both (Werfen/Immucor GTI Diagnostics) according to the manufacturer’s instructions.

##### Serum Testing for IgG Non-HLA Antibodies by the Solid-Phase Assay

Serum samples from all 74 participants were tested with the Lifecodes Non-HLA antibody kit (LNHLA) (Werfen/Immucor GTI Diagnostics) to determine the presence and specificity of IgG non-HLA antibodies according to the manufacturer’s instructions. In brief, the LNHLA assay comprises different sets of microspheres conjugated to purified recombinant or commercially available clinically significant proteins outside the HLA system. Detection is performed with a conjugate of anti-human IgG antibodies and phycoerythrin, which specifically binds to IgG antibodies from the tested serum that are already bound to the non-HLA antigens on the microspheres. The test targets 60 different non-HLA antigens and allows the identification of 60 unique IgG non-HLA antibody specificities (Supplementary Table S1). The panel includes antigens that have been shown in previously published studies to elicit post-transplant immune responses as non-HLA de novo DSA. Each antigen was conjugated to a different set of microspheres for the detection of the corresponding IgG non-HLA antibody.

### 4.3. Statistical Methods

The categorical data are presented as absolute and relative frequencies. Absolute frequencies, determined by direct counting, include the number of participants with positive and negative test results, the number of specific IgG anti-HLA and IgG non-HLA antibodies and the number of individual alloimmunization events. Relative frequencies were calculated by dividing absolute frequencies by the total number of participants in the study or the relevant subgroup. Descriptive statistics, including mean, median, minimum, maximum, interquartile range (IQR) and standard deviation (SD), were applied to summarize continuous data. Differences in numerical variables were tested using the following statistical methods: Fisher’s exact test, two-tailed t-test for two proportions, Mann–Whitney U test, Kruskal–Wallis test and point-biserial correlation coefficient. Statistical significance was set at *p* < 0.05. Data were analyzed using MedCalc Statistical Software version 22.021 (MedCalc Software, Ostend, Belgium) and SPSS Statistics 29 (IBM Corp., 2022. IBM SPSS Statistics for Windows, Version 29.0. Armonk, NY, USA: IBM Corp.).

## 5. Conclusions

This study showed a high prevalence of IgG non-HLA antibodies present in the sera of 91.9% of participants, most of whom had multiple antibody specificities. The most common non-HLA antibodies were anti-ENO1 (28.4%), anti-FIBR1 (23.0%) and anti-PRKCZ (23.0%), indicating their possible involvement in the immune responses of patients with end-stage renal disease on the kidney transplant waiting list. In addition, specific anti-HLA antibodies were identified in 43.2% of participants, with 35.1% having anti-HLA class I antibodies and 28.4% having anti-HLA class II antibodies. A significant difference was found in the number of distinct specificities between anti-HLA and non-HLA antibodies, reflecting the different immune profiles associated with these antibody types. However, no significant correlation was found between the number of non-HLA antibody specificities and previous alloimmunization events such as transfusions, pregnancy or previous kidney transplants, as well as the type and duration of dialysis treatment. This study included the entire population of 74 patients on the waiting list for kidney transplantation at the Clinical Hospital Center Rijeka who met the inclusion criteria, and not just a representative sample. Although the absolute number of patients was small, future studies with larger participant groups are needed to further validate these results. The study provides valuable insight into the dynamics of non-HLA antibody development and highlights the need for further research on the frequency of specific non-HLA antibodies and their relationship to immune status and previous immunization events. Despite limitations, this study provides a solid foundation for a more comprehensive investigation of non-HLA antibodies and emphasizes the importance of exploring their clinical significance in patients awaiting solid organ transplantation. These findings highlight the complexity of alloimmunization in transplant patients, where non-HLA antibodies can influence the immune response independently or synergistically with anti-HLA antibodies. The presence of multiple non-HLA specificities reflects the complexity of alloimmunization in these patients.

Our aim was to determine the frequency of pre-existing antibodies against these clinically significant non-HLA antigens in our patient cohort. Since there is currently no typing procedure for non-HLA antigens or alleles as there is for HLA, identifying pre-existing antibodies is crucial. This knowledge allows us to distinguish between pre-existing antibodies and de novo non-HLA DSA in post-transplant monitoring. Such differentiation may influence clinical decisions regarding immunosuppressive therapy and could potentially reduce the costs associated with retransplantation if these antibodies lead to rejection.

Given that Croatia ranks among the countries with the highest per capita kidney transplantation rates and, more generally, organ donation rates worldwide, understanding the role of non-HLA antibodies in our patient population is particularly important. While identifying non-HLA antibody specificities for prospective screening is challenging due to population differences, the availability of the commercially available panel we used makes it a valuable resource for various patient groups. The clinical implications of non-HLA antibodies are objectives for future translational research, and we trust that our findings will inform future studies and contribute to the development of improved diagnostic and monitoring strategies in transplantation.

## Figures and Tables

**Table 1:** The distribution of IgG anti-HLA-A antibody specificities in the studied population (waiting list for kidney transplantation at CHC Rijeka, Croatia).

Antigen HLA-A	Number of Participants with Specific Anti-HLA Antibody	Frequency of Specific Anti-HLA Antibody in Study Population	Frequency of Antigen-Encoding HLA Alleles in Croatian Population [41]	Comparison of Frequencies * (*p*-Value)
A1	5	6.8%	13.18%	0.1031
A2	11	14.9%	30.26%	0.0040
A3	5	6.8%	11.48%	0.2041
A11	8	10.8%	7.21%	0.2340
A23	9	12.2%	2.30%	<0.00001
A24	10	13.5%	11.79%	0.6455
A25	9	12.2%	2.87%	<0.00001
A26	6	8.1%	4.98%	0.2187
A29	4	5.4%	0.75%	<0.00001
A30	5	6.8%	1.69%	0.0009
A31	6	8.1%	2.16%	0.0005
A32	9	12.2%	4.29%	0.0009
A33	4	5.4%	2.06%	0.0444
A34	6	8.1%	0.02%	<0.00001
A36	5	6.8%	0.00%	N/A
A43	6	8.1%	0.00%	N/A
A66	6	8.1%	0.40%	<0.00001
A68	8	10.8%	4.46%	0.0088
A69	9	12.2%	0.07%	<0.00001
A74	4	5.4%	0.01%	<0.00001
A80	4	5.4%	0.07%	<0.00001

* Fisher’s exact test was used for comparison (one degree of freedom, significance set at *p* < 0.05). N/A = not applicable. Reference [41]: HLA allele frequencies in a sample of 10,000 participants.

**Table 2:** The distribution of IgG anti-HLA-B antibody specificities in the studied population (waiting list for kidney transplantation at CHC Rijeka, Croatia).

Antigen HLA-B	Number of Participants with Specific Anti-HLA Antibody	Frequency of Specific Anti-HLA Antibody in Study Population	Frequency of Antigen-Encoding HLA Allelesin Croatian Population [41]	Comparison of Frequencies * (*p*-Value)
B7	7	9.5%	7.31%	0.4777
B8	5	6.8%	8.06%	0.6818
B13	10	13.5%	3.38%	<0.00001
B14	9	12.2%	2.71%	<0.00001
B15	50	67.6%	4.88%	<0.00001
B18	4	5.4%	8.67%	0.3173
B27	9	12.2%	6.30%	0.0394
B35	5	6.8%	13.42%	0.0930
B37	9	12.2%	0.91%	<0.00001
B38	10	13.5%	4.66%	0.0003
B39	6	8.1%	2.96%	0.0096
B40	15	20.3%	3.85%	<0.00001
B41	5	6.8%	1.02%	<0.00001
B42	6	8.1%	0.00%	N/A
B44	8	10.8%	9.73%	0.7566
B45	4	5.4%	0.18%	0.0209
B46	4	5.4%	0.01%	<0.00001
B47	8	10.8%	0.14%	<0.00001
B48	4	5.4%	0.16%	0.0099
B49	10	13.5%	1.74%	<0.00001
B50	6	8.1%	1.26%	<0.00001
B51	10	13.5%	10.63%	0.4237
B52	9	12.2%	1.45%	<0.00001
B53	9	12.2%	0.40%	<0.00001
B54	6	8.1%	0.03%	<0.00001
B55	6	8.1%	1.35%	<0.00001
B56	9	12.2%	1.11%	<0.00001
B57	14	18.9%	2.54%	<0.00001
B58	13	17.6%	1.16%	<0.00001
B59	11	14.9%	0.00%	N/A
B67	6	8.1%	0.00%	N/A
B73	6	8.1%	0.05%	<0.00001
B78	7	9.5%	0.00%	N/A
B81	5	6.8%	0.00%	N/A
B82	4	5.4%	0.00%	N/A

* Fisher’s exact test was used for comparison (one degree of freedom, significance set at *p* < 0.05). N/A = not applicable. Reference [41]: HLA allele frequencies in a sample of 10,000 participants.

**Table 3:** The distribution of IgG anti-HLA-C antibody specificities in the studied population (waiting list for kidney transplantation at CHC Rijeka, Croatia).

Antigen HLA-C	Number of Participants with Specific Anti-HLA Antibody	Frequency of Specific Anti-HLA Antibody in Study Population	Frequency of Antigen-Encoding HLA Allelesin Croatian Population [41]	Comparison of Frequencies * (*p*-Value)
Cw1	4	5.4%	4.83%	0.8181
Cw2	2	2.7%	9.19%	0.0536
Cw3	9	12.2%	7.52%	0.1310
Cw4	4	5.4%	14.87%	0.0226
Cw5	1	1.4%	4.11%	0.2340
Cw6	3	4.1%	8.59%	0.1645
Cw7	5	6.8%	25.65%	0.0002
Cw8	4	5.4%	2.83%	0.1835
Cw12	5	6.8%	13.54%	0.0891
Cw14	3	4.1%	2.41%	0.3576
Cw15	4	5.4%	3.86%	0.4902
Cw16	3	4.1%	1.71%	0.1236
Cw17	2	2.7%	0.88%	0.0969
Cw18	2	2.7%	0.05%	<0.00001

* Fisher’s exact test was used for comparison (one degree of freedom, significance set at *p* < 0.05). Reference [41]: HLA allele frequencies in a sample of 10,000 participants.

**Table 4:** The distribution of IgG anti-HLA-DR antibody specificities in the studied population (waiting list for kidney transplantation at CHC Rijeka, Croatia).

Antigen HLA-DR	Number of Participants with Specific Anti-HLA Antibody	Frequency of Specific Anti-HLA Antibody in Study Population	Frequency of Antigen-Encoding HLA Alleles in Croatian Population [41]	Comparison of Frequencies * (*p*-Value)
DR1	6	8.1%	10.92%	0.4413
DR3	5	6.8%	10.61%	0.2846
DR4	3	4.1%	9.34%	0.1188
DR7	6	8.1%	8.96%	0.7949
DR8	6	8.1%	3.37%	0.0251
DR9	4	5.4%	0.24%	<0.00001
DR10	2	2.7%	0.96%	0.1285
DR11	5	6.8%	17.42%	0.0160
DR12	4	5.4%	1.56%	0.0083
DR13	6	8.1%	11.84%	0.3222
DR14	5	6.8%	3.89%	0.2041
DR15	8	10.8%	10.19%	0.9045
DR16	7	9.5%	10.74%	0.7263
DR51	6	8.1%	0.00%	N/A
DR52	5	6.8%	0.00%	N/A

* Fisher’s exact test was used for comparison (one degree of freedom, significance set at *p* < 0.05). N/A = not applicable. Reference [41]: HLA allele frequencies in a sample of 10,000 participants.

**Table 5:** The distribution of IgG anti-HLA-DQ antibody specificities in the studied population (kidney transplant waiting list at CHC Rijeka, Croatia).

Antigen HLA-DQ	Number of Participants with Specific Anti-HLA Antibody	Frequency of Specific Anti-HLA Antibody in Study Population	Frequency of Antigen-Encoding HLA Alleles in Croatian Population [42]	Comparison of Frequencies * (*p*-Value)
DQ2	1	1.4%	15.7%	0.0011
DQ4	7	9.5%	3.6%	0.0615
DQ5	5	6.8%	28.5%	0.0001
DQ6	8	10.8%	22.4%	0.0300
DQ7	4	5.4%	22.1%	0.0014
DQ8	8	10.8%	4.8%	0.0658
DQ9	7	9.5%	2.9%	0.0193

* Fisher’s exact test was used for comparison (one degree of freedom, significance set at *p* < 0.05). Reference [42]: HLA allele frequencies in a sample of 210 participants.

**Table 6:** The absolute and relative frequencies of the entire panel of the 60 IgG non-HLA antibodies along with their percentage in the total number of detected IgG non-HLA antibodies.

IgG Non-HLA Antibody	Number of Sera of the Study Participants Positive for Antibody, N	Frequency of Each Antibody Among Study Participants, % (N = 74)	Percentage of Total Number of IgG Non-HLA Antibodies, % (N = 605)	IgG Non-HLA Antibody	Number of Sera of the Study Participants Positive for Antibody, N	Frequency of Each Antibody Among Study Participants, % (N = 74)	Percentage of Total Number of IgG Non-HLA Antibodies, % (N = 605)	IgG Non-HLA Antibody	Number of Sera of the Study Participants Positive for Antibody, N	Frequency of Each Antibody Among Study Participants, % (N = 74)	Percentage of Total Number of IgG Non-HLA Antibodies, % (N = 605)
ENO1	21	28.4%	3.5%	TUBULIN	12	16.2%	2.0%	CXCL11	8	10.8%	1.3%
FIBR1	17	23.0%	2.8%	EMCN	11	14.9%	1.8%	NCL	8	10.8%	1.3%
PRKCZ	17	23.0%	2.8%	FAS	11	14.9%	1.8%	SNRPB2	8	10.8%	1.3%
P2RY11	16	21.6%	2.6%	LMNA	11	14.9%	1.8%	ACTIN	7	9.5%	1.2%
THYRO	15	20.3%	2.5%	ROR1	11	14.9%	1.8%	ARHGDIB	7	9.5%	1.2%
ATP5B	14	18.9%	2.3%	SSB	11	14.9%	1.8%	COL V	7	9.5%	1.2%
COL III	14	18.9%	2.3%	APOL2	10	13.5%	1.7%	SNRPN	7	9.5%	1.2%
DEXI	14	18.9%	2.3%	CD40	10	13.5%	1.7%	VCL	7	9.5%	1.2%
ICAM1	14	18.9%	2.3%	GDNF	10	13.5%	1.7%	VIM	7	9.5%	1.2%
STAT6	14	18.9%	2.3%	IFNG	10	13.5%	1.7%	AGRN	6	8.1%	1.0%
COL VI	13	17.6%	2.1%	IL8	10	13.5%	1.7%	IL21	6	8.1%	1.0%
CSF2	13	17.6%	2.1%	KRT8	10	13.5%	1.7%	PRKCH	6	8.1%	1.0%
GSTT1	13	17.6%	2.1%	LPHN1	10	13.5%	1.7%	SHC3	6	8.1%	1.0%
HSPB1	13	17.6%	2.1%	PLA2R1	10	13.5%	1.7%	TRANSF	6	8.1%	1.0%
MYOSIN	13	17.6%	2.1%	KRT18	9	12.2%	1.5%	TUBA1B	6	8.1%	1.0%
TUBB	13	17.6%	2.1%	LGALS3	9	12.2%	1.5%	CCP	5	6.8%	0.8%
FLRT2	12	16.2%	2.0%	PECR	9	12.2%	1.5%	HARS	5	6.8%	0.8%
GAPDH	12	16.2%	2.0%	COL I	8	10.8%	1.3%	VEGFA	5	6.8%	0.8%
LGALS8	12	16.2%	2.0%	COL II	8	10.8%	1.3%	CGB5	4	5.4%	0.7%
PTPRO	12	16.2%	2.0%	COL IV	8	10.8%	1.3%	CXCL9	4	5.4%	0.7%
								TOTAL IgG non-HLA antibodies	605	-	100.0%

**Table 7:** Distribution of the numbers of distinct IgG non-HLA antibody specificities per individual study participant.

Number of Distinct IgG Non-HLA Antibody Specificities	0	1	2–10	11–30	31–50	51–60	Total
Number of participants	6	10	43	10	2	3	74
Frequency (%)	8.1	13.5	58.1	13.5	2.7	4.1	100.0

**Table 8:** Association of the test results for IgG anti-HLA and IgG non-HLA antibodies in study population.

Participants (N = 74)	IgG Non-HLA Antibody		Total, N (%)
	Negative	Positive	
IgG anti-HLA negative, N (%)	3 (4.1%)	39 (52.7%)	42 (56.8%)
IgG anti-HLA positive, N (%)	3 (4.1%)	29 (39.2%)	32 (43.2%)
Total, N (%)	6 (8.1%)	68 (91.9%)	74 (100.0%)

**Table 9:** Comparative analysis of the number of specificities of individual IgG anti-HLA and IgG non-HLA antibodies.

Groups of Antibodies (IgG Anti-HLA, IgG Non-HLA)	Comparison of Groups by Mann–Whitney U Test	
	z-Value	*p*-Value
IgG anti-HLA class I vs. IgG non-HLA antibodies	4.39	*p* < 0.001
IgG anti-HLA class II vs. IgG non-HLA antibodies	6.68	*p* < 0.001

**Table 10:** Descriptive statistics for the number of distinct antibody specificities detected in individual participants for IgG anti-HLA class I, IgG anti-HLA class II and IgG non-HLA antibodies.

Number of Distinct Specificities Detected	Median (IQR)	Mean (SD)
IgG anti-HLA class I	0.00 (8.00)	7.27 (14.61)
IgG anti-HLA class II	0.00 (1.00)	1.62 (3.54)
IgG non-HLA (60 antigen panel)	3.00 (6.00)	8.18 (13.07)

IQR = interquartile range; SD = standard deviation.

**Table 11:** Distribution of participants by alloimmunization events and presence of IgG anti-HLA and IgG non-HLA antibodies.

Groups of Participants Stratified by the Exposure to Alloimmunization Events	IgG Anti-HLA Positive Sera, N = 32	IgG Anti-HLA Negative Sera, N = 42	IgG Non-HLA Positive Sera, N = 68	IgG Non-HLA Negative Sera, N = 6
Participants not exposed to any alloimmunization events (N = 19)	2 (10.5%)	17 (89.5%)	18 (94.7%)	1 (5.3%)
	*p* < 0.00001		*p* < 0.00001	
Participants exposed to alloimmunization via transfusion (N = 49)	25 (51.0%)	24 (49.0%)	44 (89.8%)	5 (10.2%)
	*p* = 0.8415		*p* < 0.00001	
Participants exposed to alloimmunization through pregnancy (N = 18)	13 (72.2%)	5 (27.8%)	16 (88.9%)	2 (11.1%)
	*p* = 0.0076		*p* < 0.00001	
Participants exposed to alloimmunization through both transfusion and pregnancy (N = 14)	10 (71.4%)	4 (28.6%)	12 (85.7%)	2 (14.3%)
	*p* = 0.0232		*p* = 0.0002	
Participants exposed to alloimmunization through a previous kidney transplant, i.e., HLA antigens (N = 18)	16 (88.9%)	2 (11.1%)	16 (88.9%)	2 (11.1%)
	*p* < 0.00001		*p* < 0.00001	

Fisher’s exact test =was used for comparison (one degree of freedom, significance set at *p* < 0.05).

**Table 12:** Comparison of the mean, median, minimum and maximum number of distinct IgG non-HLA antibody specificities between participants who did or did not receive a transfusion of blood products.

Transfusion of Blood Products in Patient History	Yes	No
Number of participants, N (%)	49 (66.2%)	25 (33.8%)
Mean of distinct IgG non-HLA antibody specificities	8.80	6.96
Median of distinct IgG non-HLA antibody specificities	3	3
Minimum	0	0
Maximum	59	57
Comparison of mean values of distinct IgG non-HLA antibody specificities in both groups *	*p* = 0.683	

* The Mann–Whitney U test was used for the comparison of mean values; significance was set at *p* < 0.05, N = 74.

**Table 13:** Comparison of the mean, median, minimum and maximum number of distinct IgG non-HLA antibody specificities between participants who did or did not receive a transfusion of blood products.

Pregnancy in Patient History	Yes	No
Number of female participants, N (%)	18 (75.0%)	6 (25.0%)
Mean of distinct IgG non-HLA antibody specificities	7.89	19.71
Median of distinct IgG non-HLA antibody specificities	2.5	15
Minimum	0	2
Maximum	58	57
Comparison of mean values of distinct IgG non-HLA antibody specificities in both groups *	*p* = 0.083	

* The Mann–Whitney U test was used for the comparison of mean values; significance was set at *p* < 0.05, N = 24.

**Table 14:** Correlation of the number of IgG non-HLA antibody specificities and alloimmunization events.

Alloimmunization Event	Correlation Coefficient *	Significance Level
Pregnancy	0.01	*p* > 0.05
Transfusion	−0.07	*p* > 0.05
Transplantation	0.011	*p* > 0.05

* The point-biserial correlation coefficient was calculated based on the number of IgG non-HLA antibody specificities in the serum of an individual participant. Significance was set at *p* < 0.05.

**Table 15:** Comparison of the mean, median, minimum and maximum number of distinct IgG non-HLA antibody specificities for different types of dialysis treatment.

Dialysis Procedure in Patient History	No Dialysis Treatment	Peritoneal Dialysis	Hemodialysis
Number of participants, N (%)	14 (18.9%)	15 (20.3%)	45 (60.8%)
Mean of distinct IgG non-HLA specificities	10.4	12.2	6.2
Median of distinct IgG non-HLA specificities	4.5	3	3
Minimum	0	0	0
Maximum	59	57	58
Comparison: no dialysis vs. peritoneal dialysis	*p* = 0.965		
Comparison: no dialysis vs. hemodialysis	*p* = 0.306		
Comparison: peritoneal dialysis vs. hemodialysis	*p* = 0.326		
Comparison of all three groups	*p* = 0.445		

Comparisons made using the Kruskal–Wallis test, significance set at *p* < 0.05, N = 74.

**Table 16:** Duration of dialysis treatment in groups of participants (N = 60) receiving peritoneal dialysis and hemodialysis at the time of serum sample collection.

Dialysis Type	Duration of Dialysis Treatment (in Months)					Total, N %	Mean (Months)	Median (Months)	Minimum (Months)	Maximum (Months)
	1–6	7–12	13–24	25–60	61–102					
Participants on peritoneal dialysis, N (%)	2 (3.3%)	3 (5.0%)	5 (8.3%)	3 (5.0%)	2 (3.3%)	15 (25.0%)	27.1	19	5	79
Participants on hemodialysis, N (%)	5 (8.3%)	2 (3.3%)	8 (13.3%)	23 (38.3%)	7 (11.7%)	45 (75.0%)	38.1	34	1	102
Total, N (%)	7 (11.7%)	5 (8.3%)	13 (21.7%)	26 (43.3%)	9 (15.0%)	60 (100.0%)	35.4	30	1	102

**Table 17:** Comparison of the number of distinct IgG non-HLA antibody specificities between participants who had (N = 60) and had not (N = 14; Figure 2) started dialysis treatment at the time of serum sample collection.

Comparison (*p*-Values) *	Duration of Dialysis Treatment (in Months)	1–6 Months	7–12 Months	13–24 Months	25–60 Months	61–102 Months	All Participants, N = 60
Type of dialysis treatment	Hemodialysis (N = 45)	1.000	1.000	0.598	0.983	0.782	0.602
	Peritoneal dialysis (N = 15)	0.417	0.718	1000	0.532	0.567	0.830
	All participants receiving dialysis treatment (N = 60)	0.855	0.745	0.851	0.944	0.576	0.827

* Fisher’s exact test was used for comparison (five degrees of freedom, significance set at *p* < 0.05).

## Data Availability

The raw data presented in this study are available on request from the corresponding author in an anonymized form due to privacy and legal reasons.

## Supplementary Materials

The following supporting information can be downloaded at: https://www.mdpi.com/article/10.3390/ijms252212103/s1.
