# Supplementary material for: Comprehensive Characterization of Anti-HLA and Non-HLA Antibodies in Patients on Kidney Transplant Waiting List and Evaluation of Their Impact on Alloimmunization Risk and Dialysis Treatment
Source: Int J Mol Sci. 2024 Nov 11;25(22):12103. doi: 10.3390/ijms252212103 (PMC11593988; doi:10.3390/ijms252212103)
Supplement: Supplementary file 1 [file ijms-25-12103-s001.zip › ijms-3271018-supplementary.pdf]

Appendix A

Table A1. List of the 60 non-HLA antigens included in the panel used in this study, along with their corresponding coding gene names.

Antigen	Gene Name	Antigen	Gene Name	Antigen	Gene Name
ACTIN	Actin	FAS	Fas Cell Surface Death Receptor	NCL	Nucleolin
AGRN	Agrin	FIBRONECTIN1	Fibronectin 1	P2RY11	Purinergic Receptor P2Y11
APOL2	Apolipoprotein L2	FLRT2	Fibronectin Leucine Rich Transmembrane Protein 2	PECR	Peroxisomal Trans-2-enoyl-CoA Reductase
ARHGDIB	ARHGDIB	GAPDH	Glyceraldehyde-3-phosphate Dehydrogenase	PLA2R1	Phospholipase A2 Receptor 1
ATP5B	ATP Synthase F1 Subunit Beta	GDNF	Glial Cell Derived Neurotrophic Factor	PRKCH	Protein kinase C, Eta
CCP	Cyclic Citrullinated Peptide	GSTT1	Glutathione S-Transferase Theta-1	PRKCZ	Protein kinase C, Zeta
CD40	CD40 molecule	HARS	Jo-1	PTPRO	Receptor-type Tyrosine-protein Phosphatase U
CGB5	Chorionic Gonadotropin Subunit B5	HSPB1	Heat Shock Protein Beta-1	ROR1	Receptor Tyrosine Kinase-Like Orphan Receptor 1
COLLAGEN I	Collagen I	Human Transferrin	Transferrin	SHC3	SHC Adaptor Protein 3
COLLAGEN II	Collagen II	ICAM1	Intracellular Adhesion Molecule 1	SNRPB2	Small Nuclear Ribonucleoprotein Polypeptide B
COLLAGEN III	Collagen III	IFNG	Interferon Gamma	SNRPN	Small Nuclear Ribonucleoprotein Polypeptide N
COLLAGEN IV	Collagen IV	IL21	Interleukin 21	SSB	Sjögren Syndrome Antigen B
COLLAGEN V	Collagen V	IL8	Interleukin 8, CXCL8	STAT6	Signal Transducer And Activator Of Transcription 6
COLLAGEN VI	Collagen VI	KRT18	Cytokeratin 18	Thyroglobulin	Thyroglobulin
CSF2	Colony Stimulating Factor 2	KRT8	Cytokeratin 8	TUBA1B	Tubulin Alpha 1b
CXCL11	C-X-C Motif Chemokine Ligand 11	LGALS3	Galectin 3	TUBB	Tubulin Beta
CXCL9	C-X-C Motif Chemokine Ligand 9	LGALS8	Galectin 8	TUBULIN	Tubulin
DEXI	Dexamethasone-induced transcript	LMNA	Lamin-A/C	VCL	Vinculin
EMCN	Endomucin	LPHN1	Latrophilin 1	VEGFA	Vascular Endothelial Growth Factor A
ENO1	Alpha-enolase	MYOSIN	Myosin	VIM	Vimentin
